# Glomangioma of the lung: a case report and review of the literature

**DOI:** 10.1186/1752-1947-8-5

**Published:** 2014-01-03

**Authors:** Florian Gebauer, Alexander Quaas, Jakob R Izbicki, Yogesh K Vashist

**Affiliations:** 1 Department of General, Visceral and Thoracic Surgery, University Medical Center Hamburg-Eppendorf, University of Hamburg, Martinistrasse 52, 20246 Hamburg, Germany; 2 Department of Pathology, University Medical Center Hamburg-Eppendorf, University of Hamburg, Hamburg, Germany

**Keywords:** Glomangioma, Glomus tumor, Pulmonary tumor, Thoracic surgery

## Abstract

**Introduction:**

Glomangiomas are rare soft tissue tumors originating from the perivascular tissue. The most common localization is in the dermis of the extremities, with a few reports of respiratory tract involvement.

**Case presentation:**

We present the case of a 48-year-old Caucasian female patient with a glomangioma in her left lung. It was diagnosed incidentally as a coin lesion in a chest X-ray performed during preoperative work-up for a gastric Roux-en-Y bypass for alimentary obesity. A computed tomography scan of her chest revealed a lesion in her upper left lung lobe 31mm in diameter. After resection, a histopathological examination presented typical signs of a glomangioma, originating from the pulmonary parenchyma.

**Conclusion:**

Glomangiomas of the lung are extremely rare. However, whenever incidental lesions in the lung parenchyma are found, glomangioma should be taken into diagnostic consideration. To the best of our knowledge, signs of malignancy have not previously been reported in the literature. In fact, this tumor entity shows benign behavior, with a low potential for recurrence after complete resection.

## Introduction

Glomangiomas are rare tumors typically observed in the dermis of the extremities. Very rarely, glomangiomas occur in different organs, including the respiratory system [1-3]. We present a primary glomangioma located in the lung of a 48-year-old Caucasian female patient.

## Case presentation

A 48-year-old Caucasian female patient presented with an incidental finding of a solitary pulmonary lesion in a chest X-ray during preoperative work-up for a Roux-en-Y gastric bypass for alimentary obesity. Her medical history was unremarkable, except for obesity (body mass index, 42kg/m^2^) and arterial hypertension.

**Figure 1:**
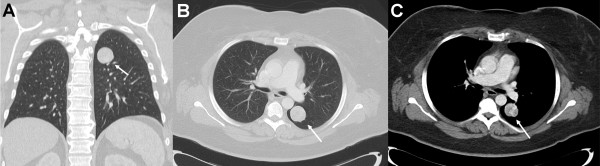
**Computed tomography of the chest. (a)** Coronal and **(b, c)** axial plane. The glomangioma is marked with an arrow.

A chest computed tomography (CT) scan confirmed the expected lesion in the upper left lobe of her lung, with a maximum diameter of 31mm in the axial plane and contrast enhancement at the outer tumor margin (Figure 1). Suspicious hilar or mediastinal lymph nodes were not detected. An ^18^fluorodeoxyglucose positron emission tomography (^18^FDG-PET) scan showed low glucose utilization (standardized uptake value, 4.46) and we did not suspect malignancy. A bronchoscopy with alveolar lavage did not show any intraluminal tumor growth and a histopathological examination revealed regular bronchial epithelial cells. Initially, our patient refused an operation to remove the glomangioma. A chest CT scan three months later showed the tumor had not changed. At this time, our patient agreed to a surgical procedure and we performed a pulmonary wedge resection of her upper left lobe via a mini-thoracotomy. Intraoperative frozen sections revealed no signs of malignancy. Exact classification of the entity was not possible from the frozen sections. There were no postoperative complications and our patient was discharged six days postoperatively.

**Figure 2:**
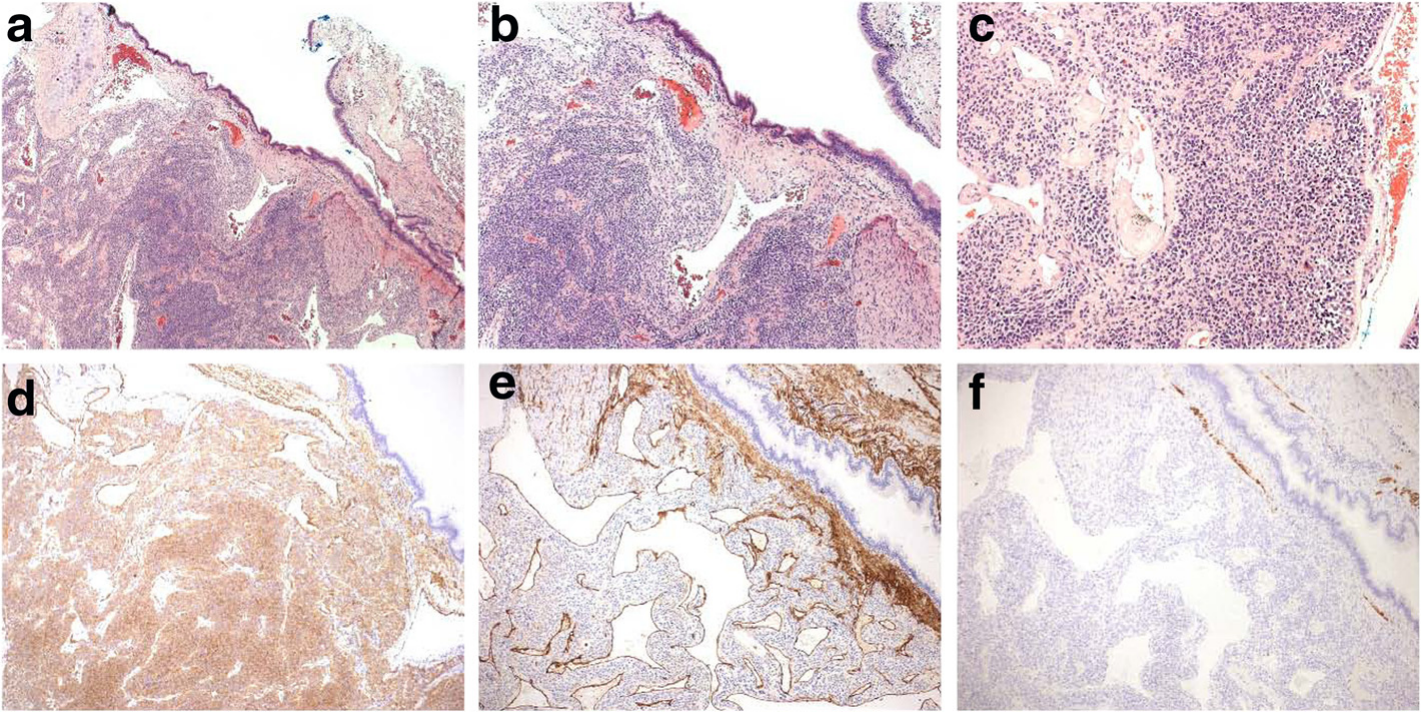
**Histological examination of the specimen.** Hematoxylin-eosin staining at **(a)** two-fold, **(b)** five-fold and **(c)** ten-fold magnification. Immunohistochemical staining for **(d)** smooth muscle antigen, **(e)** CD34 and **(f)** desmin at five-fold magnification.

The histopathological findings presented a classic glomangioma containing highly homogenous glomus cells and partially dilated blood vessels, but no signs of cellular atypia (Figure 2). The tumor measured 33mm in maximal diameter, showed contact with the wall of a large bronchus, and was surrounded by regular alveolar tissue. Immunohistochemical examinations revealed a very low proliferation rate, based on molecular immunology Borstel-1 (MIB-1) being present in less than 1% of the cells, while smooth muscle antigen was positive in the cytoplasm of almost all of the tumor cells. Type IV collagen exhibited a chicken-wire pattern between the cells, which were negative for S-100, CD31, estrogen receptor and pancytokeratin AE1/AE3 staining in the glomus cells.

## Discussion

Glomangioma are derived from glomus bodies, and comprise less than 2% of all soft tissue tumors [1,4]. Glomus bodies are arteriovenous anastomoses, which are associated with blood flow and temperature regulation.

The histopathological characteristics of glomangiomas are very close to those of glomus bodies. Both are composed of epithelioid smooth-muscle cells, which are typically arranged in sheets and nests around the blood vessels. Within the glomus tumor family, different types of tumors have been described according to their morphological presentation. The glomus tumor itself consists predominantly of glomus cells, whereas the glomangioma presents with an extremely high density of vascularity, including dilated blood vessels or cavernous blood spaces. In a histological examination, the differential diagnosis must exclude several different tumors, such as carcinoid tumors, hemangiopericytomas, sclerosing hemangiomas, leiomyomas and paragangliomas. In general, each tumor entity can be definitively identified by their specific immunohistochemical staining pattern. For example, carcinoid tumors are sometimes mistaken for glomus tumors because they have a comparable conventional histological presentation. However, carcinoid, but not glomus, tumors are positive for cytokeratin and neuroendocrine markers such as chromogranin A and synaptophysin. Hemangiopericytomas are positive for vimentin and CD34, but negative for cytokeratin and smooth muscle markers. Paragangliomas are typically composed of round epithelioid cells with small nuclei, and express neuroendocrine markers and S-100 protein.

Primary glomangiomas and glomus tumors of the lung are extremely rare - to the best of our knowledge, only 30 cases have been described in the literature [3,5,6]. The majority of these cases are reports of glomus tumors; glomangiomas in the lung have been described in only five cases [1,3,7].

The biological behavior of glomangiomas seems to be benign according to the follow-up data available. So far, follow-up data is accessible for four patients ranging from 9 to 60 months after surgery, without evidence of a relapse [1,3]. Malignant glomus tumors have been described in the literature in only six cases. Those tumors showed characteristics of a glomangiosarcoma with infiltrative growth and cellular atypia [5,8-10]. However, lymph node metastases have not been observed in any of the cases.

In our opinion, due to their uncertain biological behavior, resection of these tumors is warranted once they have been diagnosed. Preoperative imaging and biopsy seldom lead to a conclusive diagnosis, because glomangiomas do not present with specific characteristics in CT or magnetic resonance imaging, and utility of FDG-PET is also of limited value. In none of the reported cases was a correct diagnosis possible preoperatively.

Whether an anatomical lobe resection with lymphadenectomy or an atypical wedge resection is the adequate method for surgical treatment of glomangioma remains unanswered. In our opinion, the wedge resection seems to be adequate if no malignancy is seen in the frozen sections. Otherwise oncological surgical standards should be applied similar to the treatment of non-small cell lung cancer.

## Conclusion

Glomangiomas of the lung are rare tumors, and only a few cases have been reported in the literature. Even though glomangiomas are typically benign, invasive subtypes, such as glomangiosarcoma, have been reported. Glomangiomas of the lung are usually diagnosed incidentally and should always be resected as the behavior cannot be predicted by preoperative examinations. While a complete surgical resection of the tumor is mandatory, a pulmonary wedge resection should be adequate. Even though intraoperative frozen sections may help to determine tumor-free surgical resection margins, a definitive diagnosis from frozen sections is often not possible.

## Consent

Written informed consent was obtained from the patient for publication of this case report and accompanying images. A copy of the written consent is available for review by the Editor-in-Chief of this journal.

## Abbreviations

CT: Computed tomography; FDG-PET: ^18^Fluorodeoxyglucose positron emission tomography.

## Competing interests

The authors declare that they have no competing interests.

## Authors’ contributions

FG and YKV prepared the manuscript and performed clinical treatment of and surgery on the patient. AQ performed the conventional histological examination and immunohistochemical staining. JRI reviewed the manuscript and study protocol. All authors read and approved the final manuscript.
